# Vitamin D status in non-pregnant women of reproductive age: a study in Southern Thailand

**DOI:** 10.1038/s41598-023-42557-5

**Published:** 2023-09-14

**Authors:** Somchit Jaruratanasirikul, Sasivara Boonrusmee, Staporn Kasemsripitak, Tansit Saengkaew, Kanjana Chimrung, Hutcha Sriplung

**Affiliations:** 1 Department of Pediatrics, Faculty of Medicine, Prince of Songkla University, 15 Kanchanavanich Road, KhoHong District, Hat Yai, 90110 Songkhla Thailand; 2 Nutrition Unit, Faculty of Medicine, Prince of Songkla University, Hat Yai, 90110 Songkhla Thailand; 3 Epidemiology Unit, Faculty of Medicine, Prince of Songkla University, Hat Yai, 90110 Songkhla Thailand

**Keywords:** Chemical biology, Environmental sciences, Endocrinology, Chemistry

## Abstract

Vitamin D inadequacy is a global problem in all age groups. Although there are various studies of vitamin D status in pregnant women in Southeast Asia, to date there are few studies from Southeast Asia examining vitamin D status in non-pregnant women of reproductive age. To examine the prevalence of vitamin D insufficiency (VDI) in healthy non-pregnant women of reproductive age in Southern Thailand, 120 healthy non-pregnant women aged 18–42 years were enrolled. Demographic and lifestyle data relevant to vitamin D assessment (sunlight exposure, nutritional intake, type of dress, sunscreen use) and biochemical studies (serum 25-hydroxyvitamin D or 25OHD, parathyroid hormone, calcium, phosphate) were obtained. VDI was classified as serum 25OHD < 20 ng/mL. The average serum 25OHD level was 23.1 ± 6.0 ng/mL. The overall prevalence of VDI was 34.1%. The average dietary intake of calcium, phosphorus and vitamin D and the average duration of sunlight exposure per week were not significantly different between the VDI women and the vitamin D sufficient (VDS) women. Logistic regression analysis found that the significant risk factors for VDI were greater body mass index and higher family income (p-values 0.01 and 0.02, respectively). The prevalence of VDI in non-pregnant women was high at 34%. As the dietary sources of vitamin D are limited and cutaneous vitamin D synthesis is limited by avoidance of sunlight exposure, vitamin D fortification in common daily foods would be an alternative option to reach the recommended vitamin D intake generally of at least 800 IU/day.

## Introduction

Vitamin D is an essential fat-soluble vitamin that has important roles in maintaining bone health and regulating calcium and phosphate homeostasis in blood circulation^1,2^. Humans get vitamin D from 2 natural sources, dietary vitamin D intake and cutaneous vitamin D synthesis from sunlight ultraviolet B (UVB) exposure at wavelengths of 290–315 nm. The natural dietary sources of vitamin D2 are limited to only a few kinds of fungi, and vitamin D3 is naturally obtained only from some oily fish, cod liver oil, animal organs, egg yolks and mushrooms which are not part of the common daily food intake. Therefore, natural dietary intake of vitamin D is not enough to reach the recommended daily requirement in adults of at least 800 IU/day^3^. The other main source of vitamin D is cutaneous vitamin D synthesis from direct sunlight exposure. However, many factors are associated with inadequate cutaneous vitamin D synthesis including geographic latitude, season, skin type, duration of daytime outdoor activities, skin coverage by clothing and area of skin exposure to sunlight, and use of sunscreen^4^.

Vitamin D status can be determined by serum 25-hydroxyvitamin D (25OHD) concentration, the major circulating form of vitamin D in humans with a long half-life of 14–21 days. According to the Endocrine Society guideline 2011, vitamin D status is classified as vitamin D deficiency (VDD), vitamin D insufficiency (VDI) or vitamin D sufficiency (VDS) using 25OHD levels of < 20, 21–29 ng/mL, respectively^5^. VDD and VDI have been reported from various countries to be highly prevalent at 40–90% in all age groups^6–8^ and that vitamin D inadequacy is a global problem. It is estimated that more than 1 billion people worldwide are possibly affected by vitamin D inadequacy^6^. Recently, recommendations from the Global Consensus on Prevention and Management of Nutritional Rickets 2016 and the 4th International Conference on Controversies in Vitamin D 2020 proposed a revised classification of vitamin D status as VDD, VDI and VDS at serum 25OHD concentrations of < 12, 12–20 and > 20 ng/mL, respectively^9,10^.

Southeast Asia is a region close to the equator with a warm ambient temperature and little seasonal variation throughout the year. Despite abundant sunshine in this region, however various studies among pregnant women found high prevalences of vitamin D inadequacy, as defined by a 25OHD level of < 30 ng/mL, at 40–90%^11–14^. In Thailand, there have been few studies examining vitamin D status in healthy non-pregnant women of reproductive age in Southern Thailand. This study was undertaken with the aim to examine vitamin D status and the prevalence of vitamin D insufficiency among healthy non-pregnant women of reproductive age in Southern Thailand using the 2016 and 2020 classifications of vitamin D status. Secondary outcomes were to determine whether there were any correlations between serum 25OHD level, vitamin D intake and duration of sunlight exposure, and the risk factors associated with vitamin D inadequacy.

## Materials and methods

### Sample size calculation and ethics consideration

This study was performed at Songklanagarind Hospital, the major tertiary care center and a medical school hospital in Southern Thailand, as part of a project studying vitamin D levels in infants aged 6–12 months which included measuring vitamin D level in their mothers. The protocol for this study was approved by the Institutional Review Board and the Ethics Committee of Songklanagarind Hospital, Prince of Songkla University (REC.63-358-1-1).

The sample size calculation was based on the reported prevalence of vitamin D insufficiency in the Thai population of 40% with a study power of 80%. The required sample size was calculated to be 63. We expanded the recruitment to 120 to increase the power of the study to > 90%. Written informed consent was obtained from each study participant.

The study area was Hat Yai, the largest metropolitan area of Southern Thailand, 780 km (485 miles) above the equator with 5–6 sunshine hours/day, 2 seasons (summer and rainy seasons), and an average temperature of 24–34 °C all year round^15^.

### Participants

From December 2020 to November 2021, 120 healthy non-pregnant women were enrolled in this study. After enrollment, demographic data were collected including age, level of education, occupation, family income, alcohol consumption, tobacco use, current medications, and usual dress type. Weight and height were measured in the standing position in light clothes without hat, shoes or belt. The body mass index (BMI) was calculated for each subject as the weight in kilograms divided by the height in meters squared. According to the World Health Organization (WHO) classification of BMI status, the women were classified as underweight, average or obese with BMIs of < 18.5, 18.5–24.9, and > 25.0 kg/m^2^, respectively^16^.

### Sunlight exposure, dress and use of sunscreen

Each participant was interviewed for daytime sunlight exposure, usual type of dress, use of sunscreen and the skin area(s) where topical sunscreen was applied daily. The duration of sunlight exposure was assessed using a structured questionnaire about the days of sunlight exposure per week, the time of the day (before 10.00 h, between 10.00 and 15.00 h and after 15.00 h) and the duration in minutes per day of sunlight exposure. The total duration of sunlight exposure time was then calculated in minutes per week by multiplying the duration of sunlight exposure per day by the number of days exposure per week.

### 24-h food intake record

A 24-h food record was used to calculate the dietary vitamin D intake. The food record used in this study was an open-ended list to collect a variety of detailed information about food consumed the day before the scheduled date of the blood test. The participant was instructed to record every kind of food intake including the components/ingredients and amount (or portion size) of each main meal, eggs, vegetables, fruits, snacks, milk, dairy products, and juices. Medication use including multivitamins and vitamin supplements were also included in the dietary intake list. The participants were interviewed in depth about their food record, which was re-checked by a dietitian who took 10–15 min to ensure the accuracy of the food record including the types, ingredients, and amounts of each food consumed.

### Calculation of calcium, phosphorus, and vitamin D intake

Vitamin D, calcium, phosphorus and magnesium intakes were calculated based on the 24-h food record using the software program INMUCAL, the standard program for calculating the nutritional content of Thai foods, created by Mahidol University, Thailand^17^.

### Biochemical measurements

A 3-mL blood sample was taken from each participant and the serum was separated for measurement of serum 25OHD, calcium, phosphate, alkaline phosphatase (ALP), and parathyroid hormone (PTH) levels. The 25OHD level was measured using a chemiluminescence immunoassay (LIAISON, DiaSorin, Stillwater, Minneapolis, USA) with intra-assay and inter-assay coefficients of variations of 3.9 and 5.5%, respectively. The PTH level was measured by radioimmunoassay (RIA) (COBAS, Roche Diagnostics, Indianapolis, USA). Serum calcium, phosphorus, and alkaline phosphatase were measured by calorimetric assay (COBAS, Roche Diagnostics, Indianapolis, USA).

According to the 2016 Global Consensus Recommendations on Prevention and Management of Nutritional Rickets and the 4th International Conference on Controversies in Vitamin D 2020, vitamin D deficiency (VDD), vitamin D insufficiency (VDI) and vitamin D sufficiency (VDS) were defined as serum 25OHD concentrations of < 12.0, 12.0–20.0 and > 20.0 ng/mL, respectively^9^.

### Statistical analysis

All statistical analyses were performed using the R program (R Foundation, Austria available from http://www.r-project.org/foundation/main.html). The demographic data were expressed in number and percentages (categorical variables) or mean ± standard deviation and ranges (continuous variables). The vitamin D intake was calculated as mean ± standard deviation. The numbers and proportions of the participants with low and adequate serum 25OHD concentrations were compared using the chi-square test and biochemical levels were compared by t-test. Pearson correlation was used to identify the relationship between serum level of 25OHD and other biochemical variables. Significance was set at p-value of < 0.05. Multiple logistic regression analysis was used to determine the variables that were significantly associated with vitamin D inadequacy.

### Ethical approval and consent to participate

All methods were performed in accordance with the relevant guidelines and regulations. Ethical clearance was obtained from the Institutional Review Board and Ethics Committee of Songklanagarind Hospital, Prince of Songkla University. The reference number for the ethical clearance for this paper was REC 63-358-1-1. Each study participant was informed about the purpose, method, expected benefit, and risk of the study. They were also informed about their full right to not participate in or withdraw from the study at any time. Written informed consent was obtained from each study participant. All procedures performed were carried out in accordance with the ethical standards laid down in the 1964 Declaration of Helsinki and the institutional ethics guidelines.

## Results

### Participant characteristics

The average age of the 120 women was 32.6 ± 4.5 years with average weight, height and BMI of 59.5 ± 11.1 kg, 159.2 ± 5.5 cm, and 23.5 ± 4.0 kg/m^2^, respectively. As classified by BMI status, 11 (9.2%) were underweight, 71 (59.1%) were average, and 38 (31.7%) were obese. The majority of the participants were Buddhist (n = 106, 88.3%) and 14 (11.7%) were Muslim. None of our participants reported alcohol consumption or tobacco use. The demographic data of the participants are shown in Table 1.

**Table 1:** Characteristics of the 120 study women, data are shown in mean ± SD or n (%) as appropriate.

Characteristic	Mean ± SD	Range
Age (years)	32.6 ± 4.5	22–42
Weight (kg)	59.5 ± 11.1	39.3–96
Height (cm)	159.2 ± 5.5	147–172
Body mass index (kg/m^2^)	23.5 ± 4.0	15.2–38.5
Body mass index status, n (%)		
Thin (< 18.5 kg/m^2^)	11 (9.2)	
Average (18.5–25.0 kg/m^2^)	71 (59.1)	
Obese (> 25.0 kg/m^2^)	38 (31.7)	
Religion, n (%)		
Buddhist	106 (88.3)	–
Muslim	14 (11.7)	–
Education Level, n (%)		
Bachelor	93 (77.5)	–
High school	27 (22.5)	–
Occupation, n (%)		
Housemaid	29 (24.2)	
Government official	20 (16.7)	
Government employee	33 (27.5)	
Business/trader	15 (12.5)	
Company employee, worker	23 (19.1)	
Family income (Baht/month)	39,812 ± 24,486	15,000–180,000
Type of dress, n (%)		
Short-sleeved shirt and pants/skirt	67 (55.8)	–
Long-sleeved shirt and pants/skirt	46 (38.3)	–
Long dress with hat/veil	7 (5.8)	–
Time of sunlight exposure, n (%)		
Before 10.00 h	81 (67.5)	–
During 10.00–15.00 h	6 (5.0)	–
After 15.00 h	33 (27.5)	–
Duration of sun exposure per day (min)	17.2 ± 10.0	3–60
Days of sunlight exposure per week, n (%)		
6–7 days	72 (62.6)	–
4–5 days	21 (18.3)	–
2–3 days	15 (13)	–
< 2 days	7 (6.1)	–
Duration of sun exposure per week (min)	105.0 ± 73.5	5–420
Use of sunscreen, n (%)		
Never	29 (24.2)	
At face	46 (38.3)	
At face and extremities	45 (37.5)	

### Sunlight exposure

In our study, the most common time the women spent for outdoor activity was before 10.00 h (n = 81, 67.5%), followed by in the afternoon after 15.00 h (n = 33, 27.5%). The preferred time for sunlight exposure was in the early morning 07.00–08.30 h or in the late afternoon 16.30–18.00 h. Only 6 participants (5.0%) spent time for outdoor activities during 10.00–15.00 h, all of whom were company workers. The average duration of the outdoor activities was 17.2 ± 10.0 min, 6–7 days per week. The average duration of sunlight exposure per week was 105.0 ± 73.5 min. Use of sunscreen was reported by 91 participants (75.8%), 38.3% at the face only and 37.5% at the face and extremities.

### Biochemical measurements and nutritional intake

The average overall serum 25OHD level was 23.1 ± 6.0 ng/mL (range 9.4–40.9). VDD, VDI and VDS were found in 1 (0.8%), 40 (33.3%) and 79 (65.8%) women, respectively (Table 2). The average PTH, calcium, phosphate, and ALP levels were all within normal ranges. However, 6 women (5.0%) had PTH levels greater than the upper normal range of 65 pg/mL, 1 in the VDD group and 5 in the VDI group. The average calcium intake was 519 ± 389 mg, which was only 60% of the Thai recommended daily intake (Thai RDI) of 800 mg/day, while the average phosphorus intake was adequate at 832 ± 344 mg (Thai phosphorus RDI 800 mg/day). The average vitamin D intake of our participants was 0.92 ± 1.31 µg, which was far below the Thai RDI of 15 µg/day or 600 IU/day. The main dietary sources of vitamin D of our participants were from milk and dairy products (30%), egg (68%), pork and poultry (83.3%), fish (45.0%) and animal organs (5.8%). None of the participants took any medications or calcium, multivitamin or vitamin D supplementation.

**Table 2:** Vitamin D status, biochemical studies, and nutritional intake of the 120 study women, data are shown in mean ± SD or n (% as appropriate).

	Mean ± SD	Range
25OHD (ng/mL)	23.1 ± 6.0	9.4–40.9
< 12.0, n (%)	1 (0.8)	
12.0–20, n (%)	40 (33.3)	
> 20.0, n (%)	79 (65.8)	
Parathyroid hormone (pg/mL)	38.8 ± 16.8	9.6–89.1 (reference 15–65)
Calcium (mg/dL)	9.3 ± 0.37	8.6–10.2 (reference 8.5–10.5)
Phosphate (mg/dL)	3.5 ± 0.5	1.7–5.6 (reference 3.2–5.6)
Alkaline phosphatase (IU/L)	88.6 ± 29.2	43–240 (reference 40–140)
Albumin (g/dL)	4.51 0.25	3.95–5.10 (reference 4.0–5.0)
Nutritional intake		
Calcium (mg)	519 ± 389	51–2282 (Thai RDI 800)
Phosphorus (mg)	832 ± 344	40–2345 (Thai RDI 800)
Vitamin D (µg)	0.92 ± 1.31	0–8.5 (Thai RDI 5.0)
Source of dietary vitamin D, n (%)		
Milk and dairy products	36 (30.0)	–
Eggs	82 (68.3)	–
Meat (pork, poultry)	100 (83.3)	–
Fish	54 (45.0)	–
Animal organs	7 (5.8)	–

RDI, recommended daily intake.

### Vitamin D status, risk factors, logistic regression analysis

The women were divided into 2 groups according to vitamin D status, a VDI group (including 1 VDD and 40 VDI) (n = 41) and a VDS group (n = 79). Although 6 women in the VDI group had PTH levels over the upper normal level (normal range 15–65 pg/mL), the average PTH level was not different between the VDI and the VDS women due to the wide variation of PTH levels in both groups. The average serum calcium, phosphorus, ALP and albumin levels were not different between the VDI and VDS groups (Table 3). The PTH level was negatively correlated with 25OHD level, but without a significant difference (r = − 0.15, *p-value* = 0.11).

**Table 3:** Comparison of characteristics and biochemical studies according to vitamin D status, vitamin D insufficiency (VDI) and vitamin D sufficiency (VDS), data are shown in mean ± SD or n (%) as appropriate.

Characteristic	VDI (N = 41)	VDS (N = 79)	p-value
25OHD (ng/mL)	16.8 ± 2.2 (range 9.4–19.9)	26.4 ± 4.7 (range 20.1–40.9)	** < 0.001**
PTH (pg/mL)	41.1 ± 17.8 (range 11.5–89.1)	37.0 ± 16.5 (range 9.6–64.0)	0.78
Calcium (mg/dL)	9.27 ± 0.36 (range 8.6–10.0)	9.35 ± 0.37 (range 8.6–10.2)	0.14
Phosphate (mg/dL)	3.44 ± 0.41 (range 1.70–4.40)	3.58 ± 0.58 (range 2.94–5.60)	0.06
Alkaline phosphatase (IU/L)	81.8 ± 22.9 (range 12–240)	75.7 ± 30.4 (range 47–86)	0.09
Albumin (g/dL)	4.50 ± 0.28 (range 3.95–5.10)	4.52 ± 0.24 (range 4.00–5.10)	0.88
**Nutritional intake**			
Calcium (mg)	578 ± 395 (range 51–1820)	492 ± 390 (range 66–2282)	0.24
Phosphorus (mg)	840 ± 340 (range 131–1495)	849 ± 346 (range 166–2244)	0.91
Vitamin D (µg)	1.02 ± 1.16 (range 0–5.0)	0.90 ± 1.40 (range 0–8.5)	0.66
Source of dietary vitamin D, n (%)			0.24
Milk, dairy products	11 (26.8)	25 (31.6)	
Eggs	32 (78.0)	50 (63.3)	
Meat	31 (75.6)	69 (97.2)	
Fish	20 (48.8)	34 (43.0)	
Animal organs	1 (2.4)	6 (7.6)	
**Demographic and clinical data**			
Weight (kg)	62.9 ± 11.0 (range 45.2–96.0)	57.9 ± 10.2 (range 39.3–85.1)	**0.03**
Height (cm)	158.3 ± 4.8 (range 148–170)	159.5 ± 5.8 (range 147–172)	0.11
Body mass index (kg/m^2^)	24.9 ± 4.3 (range 21.1–38.5)	22.7 ± 3.7 (range 15.2–28.7)	**0.003**
Body mass index status, n (%)			**0.001**
Underweight (< 18.5 kg/m^2^)	0	11 (13.9)	
Average (18.5–25.0 kg/m^2^)	21 (51.2)	50 (63.3)	
Obese (> 25.0 kg/m^2^)	20 (48.8)	18 (22.8)	
Religion, n (%)			0.22
Buddhist	38 (92.7)	68 (86.1)	
Muslim	3 (7.3)	11 (13.9)	
Educational level, n (%)			**0.048**
Bachelor’s degree	36 (87.8)	57 (72.1)	
High school	5 (12.2)	22 (27.9)	
Occupation, n (%)			0.24
Housemaid	7 (17.1)	22 (27.8)	
Government official	10 (24.4)	10 (12.7)	
Government employee	12 (29.2)	21 (26.6)	
Business/trader	4 (9.8)	11 (13.9)	
Company employee, worker	8 (19.5)	15 (19.0)	
Family income (Baht per month)	49,400 ± 33,200 (range 17,000–180,000)	34,800 ± 16,600 (range 15,000- 100,000)	**0.002**
Type of dress, n (%)			0.7
Short-sleeved shirt and pants/skirt	21 (51.2)	46 (58.2)	
Long-sleeved shirt and pants/skirt	17 (41.5)	29 (36.7)	
Entire body covered including headscarf	3 (7.3)	4 (5.1)	
Time of the day outdoors, n (%)			0.89
Before 10.00 h	29 (70.7)	52 (65.8)	
During 10.00–15.00 h	2 (4.9)	4 (5.1)	
After 15.00 h	10 (24.4)	23 (29.1)	
Duration of sunlight exposure per day (min)	16 ± 9 (range 5–30)	18 ± 11 (range 3–60)	0.45
Days of sunlight exposure per week, n (%)			0.77
6–7 days	24 (58.5)	52 (65.8)	
4–5 days	8 (19.5)	13 (16.5)	
2–3 days	7 (17.1)	9 (11.4)	
< 2 days	2 (4.9)	5 (6.3)	
Duration of sunlight exposure per week (min)	95.0 ± 63.4 (range 14–150)	109.4 ± 78.2 (range 15–300)	0.31
Sunscreen use, n (%)			0.21
Never	8 (19.5)	21 (26.6)	
Sometimes	16 (39.0)	38 (48.1)	
Every day	17 (41.5)	20 (25.3)	
Area of sunscreen use (n = 91), n (%)			0.19
Face	15 (45.5)	31 (53.5)	
Face and arms	9 (27.3)	7 (12.1)	
Face, arms and legs	9 (27.3)	20 (34.5)	

Significant values are in [bold].

For clinical characteristics, the VDI women had significantly higher weight, body mass index, education level and family income than the VDS women with *p-values* of 0.03, 0.003, 0.048 and 0.002, respectively, while the dress type, use of sunscreen, duration of sunlight exposure per day and per week, and intakes of calcium, phosphorus, vitamin D, and dietary sources of vitamin D were not significantly different between the two groups. The average duration of sunlight exposure per week in the VDI group was shorter than in the VDS group, but without statistical significance (95.0 ± 63.4 and 109.4 ± 78.2 min, respectively, *p-value* = 0.31).

Multiple logistic regression models were used to identify the factors significantly associated with VDI. The only significant variables related to VDI in our study were BMI and family income with adjusted odds ratios of 1.46 and 1.003 with *p-values* 0.01 and 0.02, respectively (Table 4).

**Table 4:** Multivariate analysis of factors associated with vitamin D insufficiency (VDI) (< 20 ng/mL) [n = 120].

Variable	Adjusted odds ratio (95% CI)	*p-value*
Weight (kg)		0.11
≤ 60	Reference	
> 60	1.12 (0.83–1.32)	
Body mass index (kg/m^2^)		0.01
≤ 25.0	Reference	
> 25.0	1.46 (1.09–1.96)	
Family income (Baht/month)		0.02
≤ 35,000	Reference	
> 35,000	1.003 (1.004–1.005)	
Education level,		0.27
High school	Reference	
Bachelor degree	1.97 (0.60–6.59)	

## Discussion

Our study focused on vitamin D status among healthy non-pregnant women using the recent 2016 and 2020 vitamin D classifications of VDD of < 12.0 ng/mL) and VDI between 12.0 and 20.0 ng/mL. The overall prevalence of vitamin D inadequacy (VDI including VDD) in the study was 34.2%. However, recent Polish recommendations 2023 by eight Polish/international medical societies and eight national specialist consultants suggest levels of total serum 25OHD concentrations indicating vitamin D deficiency of < 20 ng/mL, suboptimal status 20–30 ng/mL, and optimal concentration 30–50 ng/mL^18^. Using the recent 2023 Polish recommendations, the prevalence of VDI in our study participants would be high, up to 70–80%. The average PTH, calcium, phosphorus, ALP and albumin levels were not different between the VDI and VDS groups. However, 6 women in the VDI group had PTH and ALP levels above the normal ranges indicating the possible activation of compensatory mechanisms by increased bone resorption to keep the serum calcium level within the normal range. The dietary intake of vitamin D was far below the Thai RDI in both the VDS and the VDI women.

The main source of vitamin D in the human body is from cutaneous vitamin D synthesis from 7-dehydrocalciferol to cholecalciferol (vitamin D3) by direct sunlight exposure which provides nearly 90% of the daily vitamin D requirement whereas dietary vitamin D from food contributes only 10%^19,20^. At least, two or three in vivo non-canonical pathways of vitamin D3 activation generating novel D3-hydroxyderivatives different from 25-hydroxyvitamin D have been identified^21–27^ leading to production of non- or low-calcemic analogs^22,23^) and of lumisterol activation^25–27^. Hence, measuring vitamin D levels based only on 25OHD may not be sufficient and vitamin D assessment should include these non-canonical metabolites. In our study, the duration of sunlight exposure per day and per week was longer in the VDS women than in the VDI women, but without significant difference. Level of education and family income were both significantly higher in the VDI group than in the VDS women, which can be explained by the fact that the women with higher education levels and higher family incomes mostly worked indoors as government or company officers and spent less time outdoors than the women with lower education levels, a finding similar to many studies done in Southeast Asian countries^12,13,28–32^. The significantly greater BMI in the VDI group than in the VDS group in our study was similar to studies from Singapore that found that pre-pregnancy BMI was significantly greater in VDI women^28,29^.

25OHD is deposited in adipose tissues. However, despite high 25OHD storage in adipose tissues in obese subjects^33^, many studies have reported significantly higher prevalences of vitamin D insufficiency in obese adults than in normal weight adults^34,35^. According to a recent meta-analysis, the prevalences of vitamin D insufficiency were 35% and 24% higher in obese and overweight adults, respectively, compared to normal weight people^36^. A molecular study on subcutaneous adipose tissue by Di Nisio et al.^37^ found impaired activity of vitamin D metabolized enzyme CYP24A1 in the adipocytes of obese subjects. Furthermore, after supplementing the obese subjects with 25OHD, there was increased expression of *CYP24a1* in the adipocytes, resulting in increased serum levels of 25OHD in the obese subjects, but not in the normal weight subjects. This evidence of impaired function of CYP24A1 in adipose tissues could explain the low serum 25OHD levels with a high prevalence of VDI in obese subjects^37,38^.

The percentage of body surface area of sunlight exposure is one of the most important factors affecting vitamin D status. Many multi-ethnic studies in pregnant women found high prevalences of VDI of 62–92% among Muslim women, which were much higher than the 8–25% in non-Muslim women. The high prevalence of VDI in Muslim Malay women was explained by the fact that Muslim women were required to cover almost their entire body outdoors^28–32^. In the present study, of the 14 Muslim women who participated, only 7 dressed with their entire body covered including their heads with a headscarf. We found no differences in either religion or dress type between the VDI and VDS women, indicating there might be other factors involved in vitamin D status such as time and duration of sunlight exposure or use of sunscreen. The combination of short duration of outdoor activity, smaller area of body surface exposed to sunlight due to full body clothing, and use of sunscreen were the associated risk factors of VDI in our women, but none reached significant differences.

Our study had some strengths and limitations. The main strength was that this study focused on 25OHD levels in non-pregnant women and that the high prevalence of VDI in the non-pregnant women was from the inadequate intake of vitamin D along with the avoidance of sunlight exposure. Second, the assessments for nutritional intake were performed by using 24-h food records. Although the 24-h food record has potential limitations from over- or under-reporting of dietary intake, we compensated for this limitation by in-depth interviews by a dietitian which we believe gave us very accurate dietary information for accurate nutritional intake calculations. The notable limitations were that the sample size in this study was calculated based on the prevalence of VDI and was too small to demonstrate the risk factors associated with VDI. Second, we did not assess the skin color of the women which might have had some effects on cutaneous vitamin D synthesis. Lastly, the serum total 25OHD level was measured by RIA, which is not as accurate as the reference method by high performance liquid chromatography (HPLC) or a liquid chromatography-tandem mass spectrometer LC–MS/MS. However, there have been many studies showing good agreement between radioimmunoassay (Liaison®) and HPLC or LC–MS/MS (r^2^ = 0.91–0.95)^39,40^.

In summary, our findings suggest a high prevalence of VDI in healthy non-pregnant women in our study area in Southern Thailand, and that the significant factors related to VDI are high family income and high BMI. As the dietary sources of vitamin D are limited and cutaneous vitamin D synthesis is limited by avoidance of sunlight exposure in Thai women, vitamin D fortification in common daily foods would be an alternative option for women to reach the recommended vitamin D intake generally of at least 800 IU/day (a 1000 IU/tablet daily or a 5000–8000 IU/tablet weekly).

## Data Availability

The data are not publicly available due to it was funded by the Southern Thailand Institute of Research and Development and some data contained information that could compromise the privacy of research participants However, The data used to support the findings of this study are available from the corresponding author (SJ) upon request.
